# Does shrub encroachment reduce foraging grass abundance through plant-plant competition in Lesotho mountain rangelands?

**DOI:** 10.7717/peerj.13597

**Published:** 2022-08-12

**Authors:** Meredith Root-Bernstein, Colin Hoag

**Affiliations:** 1 CESCO, CNRS, Musée National d’Histoire Naturelle, Paris, Île-de-France, France; 2 Center for Sustainability and Applied Ecology, Santiago, Santiago, Chile; 3 Institute of Ecology and Biodiversity, Santiago, Santiago, Chile; 4 Department of Anthropology, Smith College, Northampton, Massachusetts, United States

**Keywords:** Shrub encroachment, Rangelands, Plant-plant competition, Forbs, Livestock production, Lesotho

## Abstract

Shrub encroachment is understood to be an important problem facing rangeland ecosystems globally. The phenomenon is still poorly understood both in regard to its impacts (*e.g*., on diversity, productivity, and soil properties) and its causes. We study the impacts and causes of dwarf shrub encroachment in the highlands of Lesotho. There, shrubs have been described as indicators of generalized land degradation and soil erosion. Surprisingly, our findings show that grass abundance is not reduced by shrub abundance, but that forb abundance does decrease with shrub abundance. We suggest that not enough research has been done to examine the role of forbs in livestock diets, nor in assessing its role in plant-plant competition in grass-shrub systems. Equating shrub presence with declines in available forage may be hasty, as according to our results, grasses were not decreased by shrub expansion in this context; however, forbs are critical components of livestock diets. We propose that the role of forbs in this system should be further studied, focusing on the role that high-nutrient or N-fixing forbs could play in returning nutrients to the soil and affecting livestock grazing patterns, both of which could reduce shrub abundances and favor the establishment of a richer forb community.

## Introduction

Shrub encroachment is a form of degradation considered a major threat to global rangelands (Van Auken, 2000; Roques, O’Connor & Watkinson, 2001; Lett & Knapp, 2003; Büntgen et al., 2015; Eldridge et al., 2011). Shrub encroachment, or the increase in populations of shrubs in grasslands used for livestock production, is blamed for reductions in livestock productivity. The cause of shrub encroachment is not always clear. Globally, woody encroachment is associated with increasing CO_2_ in the atmosphere, suggesting that an increase in abundance of woody plants is driven by an increasing productivity rate and a privileging of the C3 photosynthetic pathway over the C4 pathways used by many grasses (Kgope, Bond & Midgley, 2010). How shrubs invest this productivity (*e.g*., as canopy growth, root growth, vegetative reproduction, or sexual reproduction), and how this affects grasses, however, is the key issue in understanding shrub encroachment dynamics. Although increases in shrubs biomass may sometimes mean decreases in grasses biomass, it is also possible for these two variables to be independent, or even to increase together. Woody plants and grasses can have facilitative interactions: in the silvopastoral pseudosavanna woodland of semi-arid central Chile, for example, hydraulic lift from tree roots and canopy shade increases grasses and herbs biomass, and woody cover is considered beneficial for livestock production (Olivares, 2006). Tree-grass facilitation has also been documented in savanna systems (Scholes & Archer, 1997; Callaway, 1995). The facilitation effect of trees on grasses is strongest in more arid sites (Moustakas et al., 2013) and may shift toward competition in mesic sites (Dohn et al., 2013). Shrubs have also been reported to facilitate grasses and forbs establishment after disturbance or degradation in several habitats (Maestre et al., 2009; Cipriotti et al., 2014).

Nevertheless, the issue of shrub encroachment has sometimes been understood in terms of shifting dominance in shrub-grass competition. In some cases, degradation or perturbation from livestock grazing or overgrazing is thought to allow shrubs to gain a reproductive or competitive advantage and take over the habitat from grasses, in interaction with fires that favour shrubs (Roques, O’Connor & Watkinson, 2001; Van Langevelde et al., 2003; Kraaij & Ward, 2006; Funk et al., 2018; Li et al., 2022). Loss of soil fertility and structure may also interact with fire and grazing to disfavour grasses and favour shrubs (Branson, 1985; Ward, 2005). In some systems, particularly savannas, woody species and grasses fluctuate in relative abundance over time due to fluctuations in environmental factors and disturbances (Van Langevelde et al., 2003; Bond & Midgley, 2012). In some cases, shrubs also show negative density-dependence, endogenously driving their own cycles of expansion and disaggregation with influence from disturbances and environmental factors (Wiegand, Saltz & Ward, 2006; Meyer et al., 2007). A key environmental factor is the timing and abundance of rains, which may reset competitive interactions between shrubs and grasses (*e.g.*, Wiegand, Saltz & Ward, 2006; Kraaij & Ward, 2006; Gaitán et al., 2014).

Non-grass herbs, or forbs, are not always emphasized in models and studies of shrub encroachment dynamics. Grasses are considered the primary forage of livestock, which may account for the focus on grasses abundance in the rangeland and shrub encroachment literatures. However, livestock that incorporate forbs and shrubs into their diet are able to self-medicate and show increased weight gain, due to the positive effect of physiologically active chemicals in many forb and shrub structures (Huffman, 2003; Provenza et al., 2003; Villalba & Provenza, 2007), suggesting the importance of pasture plant diversity. Nevertheless, forbs and shrubs are often considered either unpalatable or of marginal productive value. Attention to forbs in studies of rangeland degradation is also justified from a community ecology perspective. Shrubs often facilitate annual forbs, particularly in more arid habitats (Rodríguez-Echeverría & Pérez-Fernández, 2003). At the same time, grasses and forbs can be in competition (Suttle, Thomsen & Power, 2007), and this competition can also be mediated by grazing and environmental factors (*e.g.*, Archer, Garrett & Detling, 1987; Madrigal et al., 2011; Hallett et al., 2019).

In the Lesotho highlands the age-old topic of shrub encroachment and whether it leads to competition or facilitation with grasses and forbs merits re-examination due to important economic interests in shrub management (Hoag, 2022). The highlands are a natural grassland-shrubland system (Scott, 1982; May, 2000; Fitchett et al., 2017) and a regional center of plant endemism (Carbutt, 2019). Occupying an elevational range between 2,200–3,482 masl, the sub-alpine and alpine zones of the highlands form part of the Maloti and Drakensberg Ranges. The primary land use in the sub-alpine and alpine zones is livestock grazing of cattle, angora goats, and merino sheep. Livestock producers follow a transhumant system with four months of summer (January–April) grazing in the alpine zone (>2,900 masl) and the remainder in the subalpine (2,290–2,900 masl) and lower-lying areas (2,000–2,290 masl) of the Senqu River Valley where settlement is primarily located.

Based on CH’s personal observations during field research between 2014 and 2019 (see Hoag, 2018, 2019), there is widespread concern among conservation workers in Lesotho surrounding shrub encroachment into areas dominated by grasses and forbs. The attribution of high importance to the shrub encroachment problem is motivated by the construction of large dam reservoirs downstream called the Lesotho Highlands Water Project, through which water is exported to South Africa. Conservation workers in government and foreign non-governmental organizations (NGOs) explain that trampling and overgrazing of grasses by livestock can cause poor water infiltration, exacerbated and indicated by the proliferation of shrubs, leading subsequently to sheet and gully erosion and ultimately sedimentation of the reservoirs of the Lesotho Highlands Water Project.

Approaches to reducing these problems have two foci. One is focus is on livestock grazing. This has resulted in pilot efforts to reorganize the livestock grazing regime according to Savory method principles. A second focus is shrubs. Shrubs cause erosion by competing with and reducing grasses cover, which in turn permits erosion in the bare-soil inter-shrubs spaces (*e.g.*, Wainwright, Parsons & Abrahams, 2000; Turnbull et al., 2008; Manjoro, Kakembo & Rowntree, 2012; Brazier et al., 2014). Shrubs-grasses competition is also thought to underlie loss of forage quality on rangelands. Concerns about rangeland erosion due to shrubs-grasses competition have resulted in government-sponsored shrub removal programmes called *fato-fato*, in which local people are paid to remove all the shrubs from entire mountainsides, leaving them piled in heaps forming contour lines. Shrub removal, along with heaps of dead shrubs forming contour lines, is expected to reduce erosion, although there do not appear to be studies assessing the outcomes of shrub removal. The erosion concern and the forage problem are both often combined and considered as a general condition of rangeland degradation (*e.g.*, Belayneh & Tessema, 2017). There has been little or no research done in the Lesotho highlands to establish whether shrub cover negatively impacts elements or indicators of rangeland degradation, whether grazing or some other factor such as plant competition, climate, or fire is the primary cause of local degradation, and whether shrub abundance negatively affects soil-water infiltration or surface water flow causing erosion in that region. However, before investing in testing this entire set of interactions with large-scale, long-term monitoring data, we propose to quickly and simply test one of the key underlying assumptions that underlies the idea that shrub encroachment results in increased erosion in the Lesotho highlands, notably the assumption that local shrubs compete with and replace grasses leaving bare soil susceptible to erosion.

In this article, we test the widespread assumption that a historical increase in shrubs in Lesotho mountain rangelands has resulted in a decrease in grasses abundance due to shrubs-grasses competition. In our first study, we focus on explaining three variables related to shrubs-grasses interactions. First, we ask whether shrubs reduce grasses presence. Second, we ask whether fire, grazing and rainfall affect the frequency of shrubs-grasses coexistence. Third, we ask whether shrubs increase surface soil water content. Finally, in a second study, we examined the response of forbs to shrub removal in order to understand the relationship between shrubs and forbs.

## Methods

### Study 1

**Figure 1:**
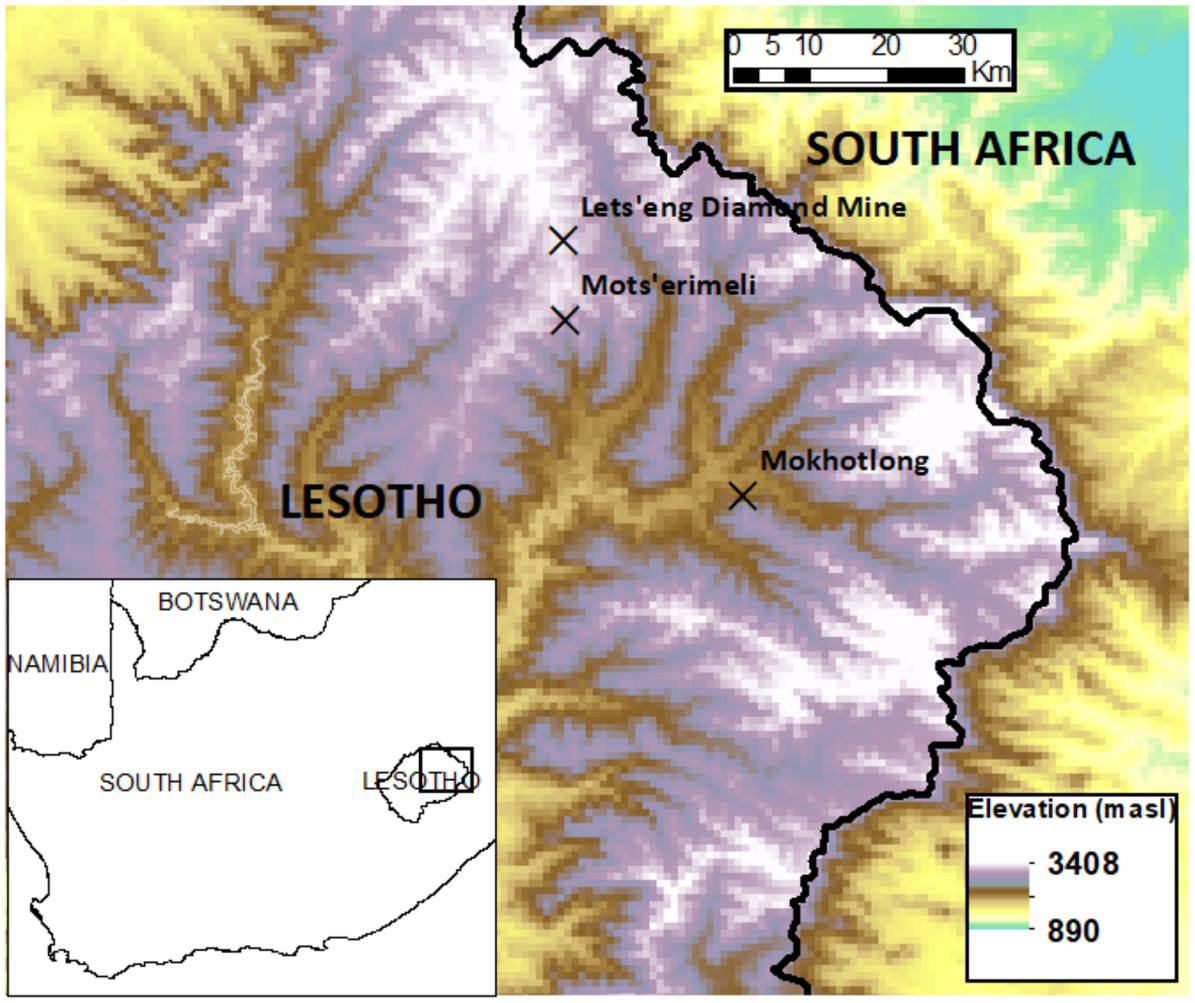
Map of Lesotho indicating the principal localities where the study took place.

Sites. In January (mid-summer) of 2019 we sampled *n* = 19 sites in and around Mokhotlong, Motšerimeli and the Letšeng Diamond Mine (Fig. 1). Lesotho’s climate is mesic according to average annual rainfall at decadal-scales, but strongly seasonal with 85% of precipitation falling between October and April and temperatures ranging from 30 °C in the summer to −5 °C in the winter (Chakela, 1999). The country also experiences regular drought as a result of the El Niño Southern Oscillation (ENSO). Our sites are in the zone where CH’s previous research took place (Hoag, 2018), facilitating negotiation of site access through personal contacts, and increasing our background knowledge of site conditions. Sample sites were chosen to represent the widest range possible of different management conditions present in the area, including variations in grazing intensity and frequency at different elevations, areas with and without recent fires, and without or at different times since deshrubbing management. Sites are shown in Table 1.

**Table 1:** Sites where data was collected, and their descriptions.

Site	Location	Elevation (m)	Condition notes
1	Airstrip, Mokhotlong	2,186	Some grazing
2	Outside airstrip, Mokhotlong	2,186	Significantly higher grazing than inside airstrip
3	Cemetery, Mokhotlong	2,149	No grazing supposedly, one cow pat
4	Behind the FTC, Mokhotlong	2,128	Little or no grazing, some old horse or donkey faeces
5	Across fence behind the FTC, beyond the horse grazing area	2,128	Some grazing
6	Lesotho Defence Force fenced area, Mokhotlong	2,096	Horse grazing
7	Across fence behind the FTC, near the Lesotho Defense Force area	2,096	Infrequent grazing by sheep, cattle, horse, donkey
8	Lower Motšerimeli	2,709	SW-facing, burned 2014, subsequently deshrubbed. Grazed by cattle, donkey, horse, sheep, goats
9	Lower Motšerimeli	2,650	Same mountain slope as above, unburnt, deshrubbed in 2013
10	Lower Motšerimeli	2,604	Same mountain slope as above, by road. Little evidence of grazing
11	Letšeng Diamond Mine	2,997	ungrazed
12	Upper Motšerimeli	2,729	E-facing, deshrubbed in 2018 approx., evidence of grazing
13	Upper Motšerimeli	2,761	W-facing, grazed 1 month/year
14	Upper Motšerimeli	2,770	W-facing, deshrubbed 1–2 months ago (2019), grazed 1 month/year
15	Upper Motšerimeli	2,730	Deshrubbed 2017, grazed 1 month/year
16	Upper Motšerimeli	2,550	W-facing, deshrubbed 2013, grazing 1 month/year
17	Mokhoaba-Motšo	2,640	NE-facing, burnt, grazed
18	Along the road from Mokhotleng-Malfiloane	2,160	Heavily grazed
19	Next to Letšeng Diamond Mine	2,961	Grazing by cattle, horses, donkeys, sheep, goats

**Figure 2:**
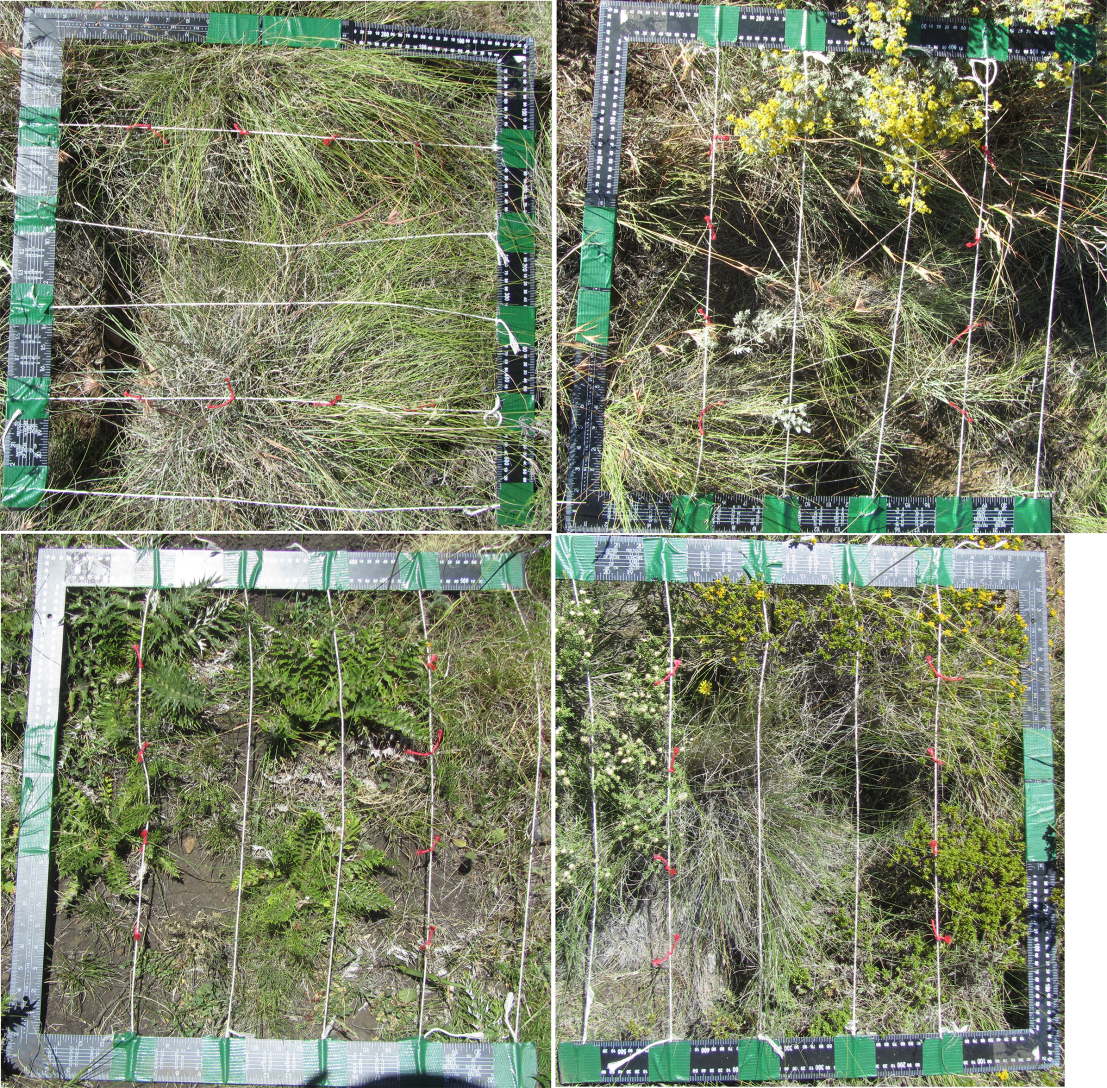
Four examples of the quadrat placed over plants during sampling. Photographs were for documentary purposes and were not used in the analysis. Some of the points along the strings are marked in red knots, and others are marked in black ink, which are hard to see in the photographs but were visible in field conditions. Not all of the strings have been straightened as they would be for data collection.

At each site, we took subsamples in the form of quadrats. At each site, we collected data from 3–10 quadrats (mode: 6) placed arbitrarily by throwing the quadrat a short distance in a pseudo-randomly chosen direction. The number of samples was proportional to the area of each site and to the observed heterogeneity (Shiyomi, Takahashi & Yoshimura, 2000; see below for explanation), which we ranked either “low”, “average” or “high”. In “low” and “average” heterogeneity sites, each sample was on average 15 m apart, and ranged from 3 to 6 quadrats. In “high” heterogeneity sites, samples were on average 10 m apart and included between six and 10 quadrats. The quadrat was a physical frame 55 × 55 cm crossed by one set of horizontal strings marked to indicate the internal points (see Fig. 2). For each quadrat we collected data at 20 grid points, in the form of a 4 × 4 interior grid (16 points) plus the four interior corners. At each point in the quadrat we recorded the presence, height and species of shrubs and grasses; forbs presence; and cover of leaf litter or bare soil. We were able to accommodate dwarf shrubs either by (1) slipping the frame under the shrub branches at ground level, or (2) holding the frame horizontally at grasses level within the shrub, with the branches inserted between the strings (maintained taught to preserve point positions) where necessary. When there was both a shrub and a grass at a point on the quadrat, we recorded an instance of shrubs-grasses coexistence. By “point” we mean anywhere along an invisible line extending above and below the actual point in the quadrat. We recorded a single layer of overlap (*i.e.*, we did not divide overlap in space into basal and canopy layers) since the shrubs are dwarf shrubs and of approximately the same size range as the grasses and forbs. In two corners of each quadrat we also measured soil hardness at the surface using a penetrometer, soil volumetric water content, soil electrical conductivity, and soil temperature, all also at the soil surface. For each site we also recorded altitude, slope aspect, grazing regime, fire recency, and shrub removal recency (the last three variables were obtained from consulting local users in the context of the semi-structured interviews described below). All quadrat data were then combined to the site level in the treatment of the data (see details below).

### Study 2

In addition, on a mountain slope where shrubs had recently been removed at Motšerimeli, we collected data on shrub and herb species richness. We chose this site because we had information about the dates of shrub removal, unlike at other sites. Shrub removal is a government-organised activity over which we have no control or influence, and we thus used the site where shrubs had been removed as a natural experiment. We thus cannot control for the fact that shrubs were removed from the lower end of the slope prior to the upper part (the people removing the shrubs start at the bottom of the slope and work up to the top of the mountain). Altitude, distance to the road, and time since shrub removal are confounded. However, given that the altitude difference across our sampled areas is only around 200 m, any difference in the flora observed is unlikely to be due to different altitudinal distributions. The constant flow of people walking along the road and all over the mountain slope to carry out the shrub removal itself also makes it relatively unlikely that distance to the road is a variable affecting the observed flora differently across our sampled areas (assuming that distance to the road would be a proxy for dispersal of exogenous seeds, which should have been equally dispersed across the sampled areas by shrub removers themselves).

We placed the same quadrat as used in Study 1 at seven arbitrary points by throwing the quadrat a short distance in a pseudo-randomly chosen direction, moving downslope in an area where shrubs had been removed 1–2 months previously. We then placed the quadrat at eight arbitrary points moving downslope to the area just above the road (and the bottom of the valley), where shrubs had been removed approximately 4–5 months earlier. The number of samples is proportionate to the area of each section such that samples were on average 13 m apart. In this study we did not observe significant differences in vegetation heterogeneity across the mountain slope, so we did not adjust the sampling density to vegetation heterogeneity. In this study, in each quadrat we ignored the interior points, and simply counted the number of species or morphospecies (for shrubs and herbs) or taxa (for mosses and succulents) within the quadrat frame.

*Justification of the sampling methods used in Studies 1 and 2*

Our quadrat scale of 55 × 55 cm was chosen to include both small dwarf shrubs or parts of larger dwarf shrubs, along with a statistically sufficient number of grasses and forbs in each quadrat, allowing us to adequately sample the interactions between shrubs, grasses, and forbs. The interior points used in Study 1 were 11 cm apart in a grid, and around 15 cm apart on the diagonal, a scale that ensured that we did not sample the same plant-plant interaction (between the same two individuals) more than once. At the same time, particularly large plants (*e.g.*, in this context, dwarf shrubs) may have particularly large plant-plant impacts, so one can consider that multiple measures of the same plant in interaction with other plants represents a weighting of its impact according to its area. This implicit weighting is appropriate because differences in biomass between interacting plants can affect the strength of competition and facilitation effects (*e.g.*, Guo et al., 2019; Trinder, Brooker & Robinson, 2013). Although plant-plant competition or coexistence is a complex and dynamic issue that cannot be entirely understood from snapshot data (Trinder, Brooker & Robinson, 2013), and different patterns of community structuring may be observed at different scales (Maestre et al., 2009; Guisan & Thuiller, 2005; le Roux et al., 2013), we chose this spatiotemporal scale of sampling because we were interested in understanding whether shrubs, grasses and forbs, on average across different life phases and conditions, are spatially mutually exclusionary at a scale relevant to livestock foraging effects (*e.g.*, Molnár et al., 2020) and shrub-herb interactions such as canopy effects (Soliveres et al., 2014).

In Study 1 we chose to sample proportionally to both site size and observed vegetation heterogeneity in order to account for important ecological interactions captured by vegetation heterogeneity. Spatial heterogeneity of underlying biophysical conditions as well as plant species autocorrelations (*i.e.*, single-species patches) can affect plant-plant interactions through spatial adjacency effects and impacts on biomass (*e.g.*, Tsutsumi, Itano & Shiyomi, 2007; Shiyomi, Takahashi & Yoshimura, 2000). To account for this, we adjusted the number of sample replicates at each site, as described above, depending on a visual approximation of the heterogeneity of the site (the vegetation was around 1 m high at most and almost always on a slope, making visual approximation highly feasible) and the area of the historical disturbance (*e.g.*, a burned area, a fenced area) whose effect we wanted to sample. We visually approximated the patchiness or homogeneity of dwarf shrubs, grasses and forbs, in terms of individual distributions and biomass differences at a 10 s of m^2^ scale, and in terms of species richness at a m^2^ scale, as recommended as the most efficient and effective method by Shiyomi, Takahashi & Yoshimura (2000). We used a visual approximation because quantification is time-consuming and often requires destructive sampling which might have affected subsequent data collection, as recommended by Patil & Stiteler (1973) and Shiyomi, Takahashi & Yoshimura (2000).

We chose to measure surface Soil Volumetric Water (VWC) because it is a measure of water available in the surface layer of the soil, which may be an ecologically relevant variable for understanding vegetation cover patterns. Surface VWC declines under evapotranspiration from plant cover, and *via* infiltration to lower soil levels, but can also rise as water is drawn up *via* evapotranspiration from lower soil levels (Wang et al., 2021). Higher values can represent better conditions for leaf litter decomposition and seed germination, and reduce litter flammability (Brant et al., 2006; Niu et al., 2021; Zhao et al., 2021). In this semi-arid habitat, surface VWC may also indicate patches of less-hydrophobic soils where rainfall can be rapidly absorbed, and if spatially non-uniform, may be an indicator of deeper soil water not immediately lost to evapotranspiration (Francis et al., 2007).

Statistical analysis. For the analysis of Study 1, focusing on shrubs-grasses competition, all data was first combined to the quadrat level, to reduce any pseudoreplication from multiple measures of interactions between pairs of large plants or non-genetic independence of clusters or tussocks of grasses. The counts of shrubs-grasses and shrubs-forbs overlap at quadrat points were summed per quadrat, and the height and presence data were respectively converted into mean heights and percentages (percent of points per plant category). Means of these data were then calculated from the sample replicates (the 3–10 quadrats per site), at the site level, which is the level at which different disturbance and management histories are relevant, and only the site-level data was included in the statistical analysis presented below. The site-level means of VWC, soil temperature, EC, and the penetrometer reading were also calculated from the quadrat-level measurements. Elevation and aspect were site-level variables. Finally, we codified information relating to disturbance and management history as categorical variables at the site scale. Grazing was codified according to the empirical observation of kinds of grazing, which included none, spring only, heavy, irregular, regular; recently burned was codified as 1 (yes) or 0 (no), and shrub removal was codified as 1 (yes) or 0 (no). The time since shrub removal was too heterogeneous, and in some cases, not known with sufficient precision, to be taken into account in this model.

We constructed a proxy variable to measure grasses biomass to test hypothesis 1. Direct measures of biomass are destructive and the use of nondestructive approximations is more efficient and recommended (Tsutsumi, Itano & Shiyomi, 2007). We used the best-performing indirect biomass estimate reported by Axmanová et al. (2012), which corresponds to multiplying grasses presence × mean grasses height at the quadrat level, averaged to the site level.

For analysis of Study 1 we first examined a set of biologically plausible correlations between all the variables included in the raw data as well as the proxy variables. We normalized the distribution of the grasses biomass proxy by taking the square root. We also normalized the distribution of number of shrubs-grasses coincidence spots per quadrat averaged to site level, using the square root. Following this, we used linear models, implemented with the *lm* command in R version 3.3.3 (R Development Core Team, 2017). We chose the best model on the basis of maximizing the number of significant variables, the overall model significance, and the F value. Not all of the variables collected in the field were included in this best model.

For the analysis of Study 2, focusing on species richness on the site where shrubs had been removed, richness was calculated by counting the number of species or morphospecies/taxa, and analysed at the quadrat scale. We calculated species richness for each quadrat as well as species turnover from the more-recently to the less-recently deshrubbed areas, and we plotted a taxon accumulation curve. Species turnover between the more-recently (the first seven samples) and less-recently deshrubbed areas (the last eight samples) was defined as (spp lost + spp gained)/total spp following Anderson (2007).

Interviews. Semi-structured interviews with experts in Lesotho (*n* = 10) were conducted based on their roles at conservation agencies active in the area of field research. Interviews with herders and livestock owners (*n* = 15) were also semi-structured, selected either because they were present in the field while vegetation sampling took place or because they volunteered to speak to us after an open-air meeting of a local grazing association. Additionally, we observed numerous conversations among experts, as well as between experts and livestock owners. Interviewees were informed orally of the purpose of our research, and the voluntary nature of the interview. The interview material is anonymized. We do not report on or analyse this material separately in this article, but we report on its use for reasons of ethics and repeatability. These interviews partially informed our study design (choice of sites for Studies 1 and 2, as described above), provided us with data on site uses, and informed interpretation and contextualization of results. Where relevant, we summarise contextualizing information we obtained from them in the Discussion.

## Results

### Study 1

*Do shrubs reduce grasses presence?*

**Figure 3:**
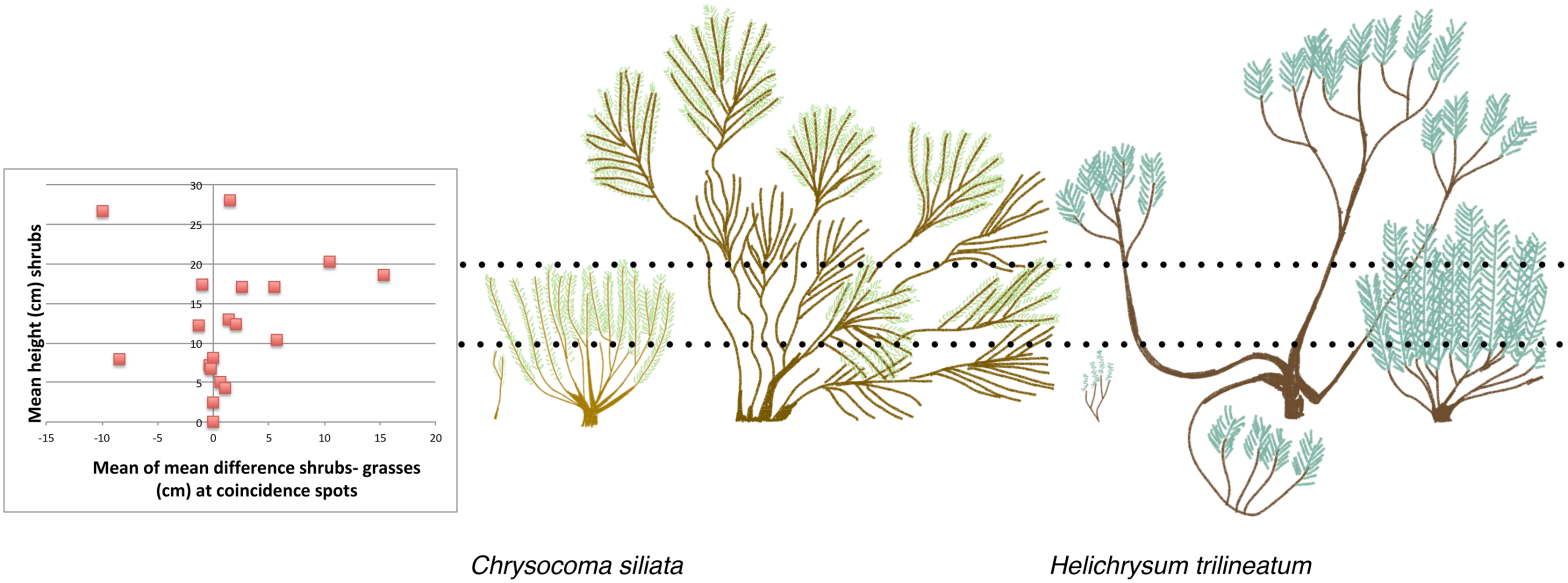
The relationship between mean shrub height and the height of grasses growing at the same spots, compared to field sketches of shrub architecture of two of the most common shrubs.

Correlations suggested *a priori* that shrubs and grasses are not in competition. If shrubs outcompeted grasses, then as the number of spots with shrubs increases, the number of spots with grasses should decrease. We found very low support for this correlation (Pearson’s correlation = 0.17). On the other hand, we did find a trend suggesting that as number of spots with shrubs increase, number of spots with forbs decrease (Pearson’s correlation = −0.36). If shrubs do not compete with grasses then as the number of spots with shrubs increases, the number of shrubs-grasses coincidence spots should increase. We found a very high correlation between the mean number of shrubs-grasses coincidence spots and the mean number of shrub spots (Pearson’s correlation = 0.83). It was also not clear that shrubs suppress grasses growth where they co-occur. We found that shrubs up to 10 cm tall were approximately the same height as the grasses with which they co-occur, while at higher shrub heights shrubs mainly overshadow grasses. This corresponds to a shift in shrub morphology for the most common shrub species at our site, from a narrow vertical shape to a dense bowl shape, which occurs around 10 cm (Fig. 3).

*Do fire, grazing and rainfall affect the frequency of shrubs-grasses coexistence?*

In the best linear model (see Methods for model selection criteria) explaining shrubs-grasses coexistence, the number of coexistence spots increased with shrub height (Table 2, adjusted R^2^ = 0.78, F_3,15_ = 15.22, *p* = 8.05 × 10^−5^). There was also a marginally non-significant positive effect of soil volumetric water content. We also looked at grasses biomass alone, since this is important for herders, and because we found above that grasses and shrubs are not in competition. The best linear model explaining grasses biomass included a significant effect of grazing regime (Table 3, adjusted R^2^ = 0.72, F_5,13_ = 10.16, *p* = 0.0004). Grasses biomass was higher in areas with no or irregular grazing. There was a non-significant positive effect of soil volumetric water content on grasses biomass.

**Table 2:** Linear model of shrub-grass coincidence spots.

	Estimate	Std. error	t value	*p* value
intercept	−0.82	0.55	−1.49	0.15
shrub height	0.06	0.02	3.17	0.006
shrub number	0.07	0.07	0.95	0.35
mean VWC	0.28	0.14	1.95	0.07

**Note:**

VWC, volumetric water content. Residual standard error: 0.4315 on 15 degrees of freedom, Multiple R-squared: 0.7527, Adjusted R-squared: 0.7033, F-statistic: 15.22 on 3 and 15 DF, *p*-value: 8.051e−05.

**Table 3:** Linear model of grass biomass.

	Estimate	Std. error	t value	*p* value
intercept	7.17	3.68	1.95	0.07
irregular grazing	9.99	2.16	4.64	0.0005
none grazing	12.83	2.58	4.96	0.0003
regular grazing	1.91	2.16	0.88	0.39
spring grazing	4.48	2.23	2.02	0.06
mean VWC	−0.06	0.78	−0.09	0.93

**Note:**

VWC, volumetric water content. Residual standard error: 2.576 on 13 degrees of freedom. Multiple R-squared: 0.7962, Adjusted R-squared: 0.7178, F-statistic: 10.16 on 5 and 13 DF, *p*-value: 0.0003916.

*Do shrubs increase surface soil water content?*

**Figure 4:**
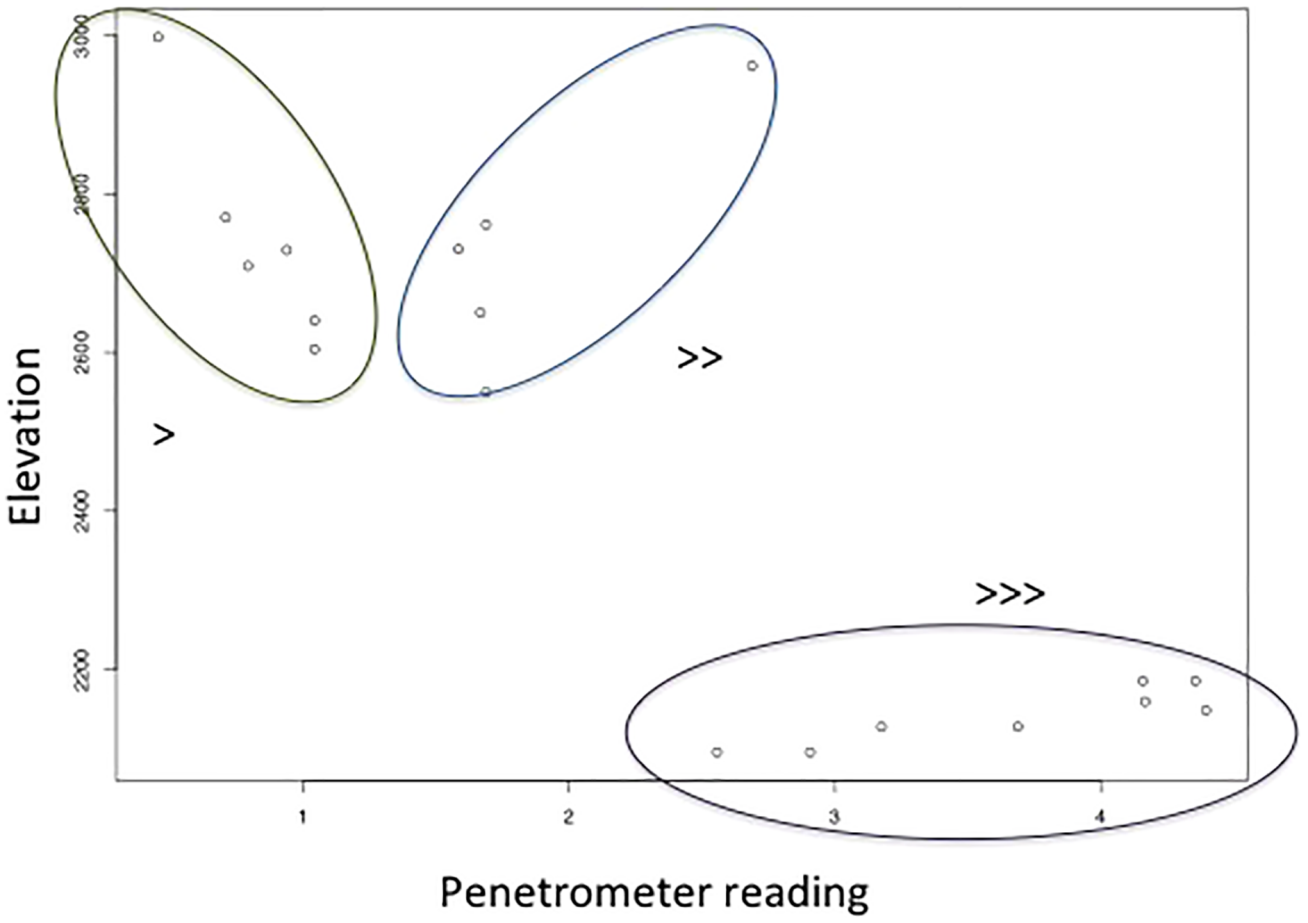
The relationship between soil hardness (increases with higher penetrometer reading) and elevation, which along with shrub number explains soil volumetric water content.

Surface soil volumetric water content increased with shrub cover (site mean of number of points occupied by shrubs in quadrats), and decreased with the interaction between elevation and soil hardness (Table 4, adjusted R^2^ = 0.46, F_4,14_ = 4.83, *p* = 0.0117). The interaction between elevation and soil hardness is highest for high soil hardness and low elevation, and lowest for low soil hardness and high elevation (Fig. 4). In other words, there was most surface soil moisture in high elevation, soft soils.

**Table 4:** Linear model of volumetric water content.

	Estimate	Std. error	t value	*p* value
intercept	−1.14	0.98	−1.16	0.26
shrub number	0.06	0.02	3.49	0.004
mean soil hardness	0.89	0.33	2.69	0.02
elevation	0.00	0.00	2.32	0.04
mean soil hardness x elevation	−0.00	0.00	−2.53	0.02

**Note:**

Residual standard error: 0.1459 on 14 degrees of freedom. Multiple R-squared: 0.58, Adjusted R-squared: 0.46, F-statistic: 4.833 on 4 and 14 DF, *p*-value: 0.01167

### Study 2

**Figure 5:**
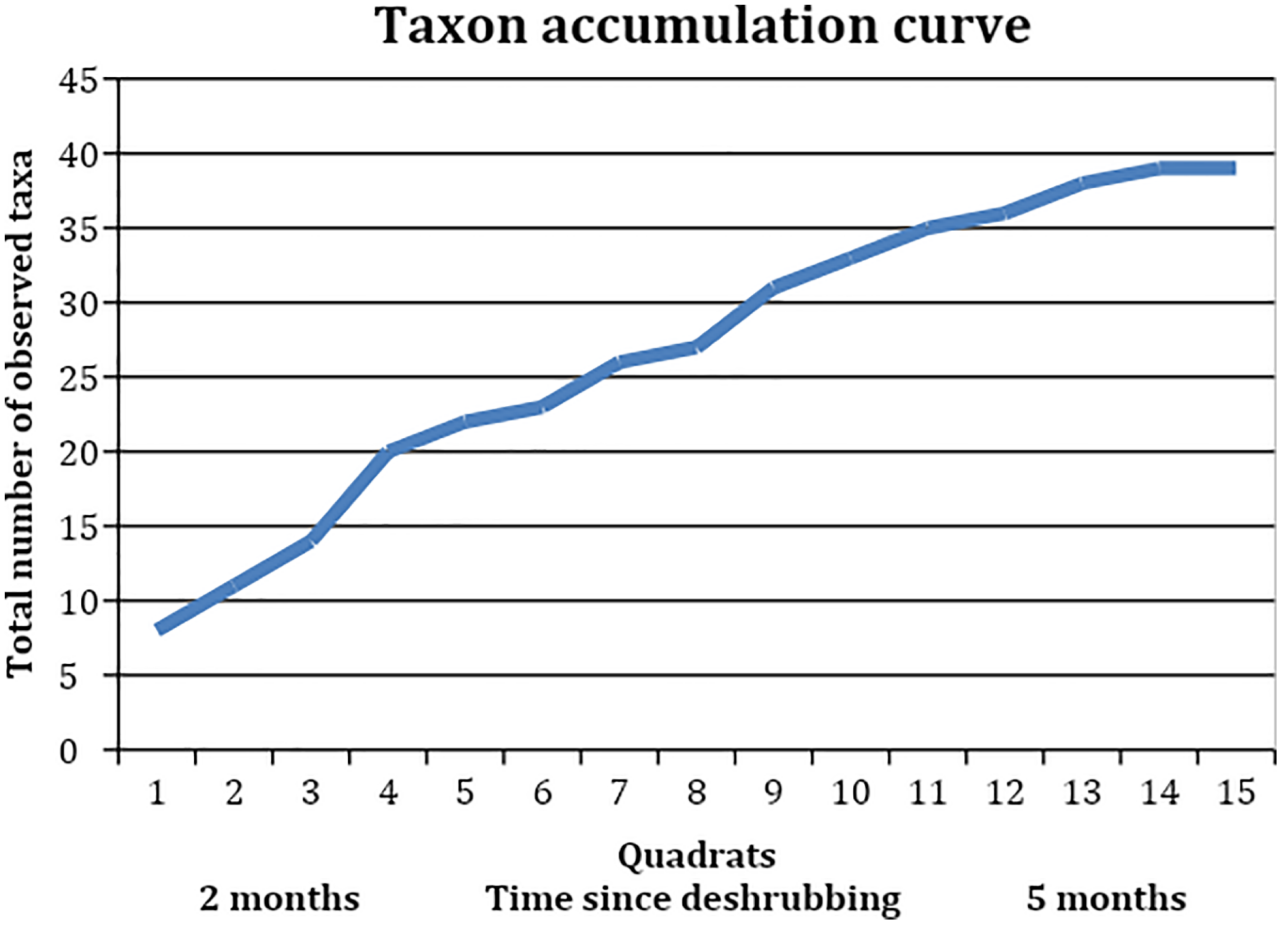
Taxon accumulation curve, showing the accumulation of observed taxa in the quadrats, which were collected in reverse order to the areas’ deshrubbing.

When looking at the richness of forbs and other species on a slope where shrubs had been removed, we found a total of 39 taxa (Table 5). These included shrubs and grasses but were dominated by forbs. The mean species richness per quadrat was almost constant between the section where shrubs had been removed 3 months ago and the section where shrubs had been removed 5 months ago, at 10 species and 10.125 species respectively (t = −0.09, df = 9.87, *p*-value = 0.92). The species turnover between these two sections was calculated as 0.54, with a loss of eight species and a gain of 13, indicating that just over half of the species making up the local plant community were different with an additional 2–3 months of time since shrub removal. The taxon accumulation curve (Fig. 5) suggests that the number of new species establishing in the areas where shrubs had been removed might begin to level off around 3 months after shrub removal, but this is unclear as it simply corresponds to the longest time since shrub removal available at that site.

**Table 5:** Plant species found in the quadrats on a deshrubbed slope.

Found only in 5 mo. since deshrubbing section	Found in both sections	Found only in 2 mo. since deshrubbing section
Un-ID’ed forb 1	*Bromus* sp. (*B. speciosa, B. catharticus*)	*Gazania krebsiana*
lichen	*Aristida spp. (A. congesta, A. diffusa, A. junctiformis*)	*Fingeruthia sp*.
*Eragrostis* spp. (*E. chloromelas, E. plana, E. curvula*)	*Oxalis depressa*	Un-ID’ed forb 8
Un-ID’ed forb 2, yellow flower	*Selago* sp.	cosmopolitan Asteracea in rosette form 2
moss	*Berkheya cirsifolia*	cosmopolitan Asteracea in rosette form 3
*Diasca ovata*	*Chrysochoma ciliata*	Un-ID’ed forb 9
Un-ID’ed forb 3, creeping habit	*Cotula spp*.	mat shrub
*Helichrysum sessiloideds*	Un-ID’ed forb 6	*Delosperma hirtum*
Un-ID’ed forb 4	sedge	Un-ID’ed forb 10
Un-ID’ed forb 5, blue flower	*Senecio sp. (purple flower)*	
*Berkheya* spp.	*Scabiosa columbaria*	
*Senecio* sp. (white flower)	*Helichrysum trilineatum*	
*Wahlenbergia cuspidata*	*Themeda triandra*	
	*Festuca caprina*	
	cosmopolitan Asteracea in rosette form 1	
	*Helichrysum* sp. (*basalticum*?)	
	Un-IDed grass	
	Un-ID’ed forb 7	

**Note:**

Unfortunately we lost the specimens intended for identification and so we were not able to fully identify all of the observed species or morphospecies.

## Discussion

Although many claims are made about the ecology of the Lesotho highlands rangelands based on research in other study systems, this modest empirical study is one of the only site-specific pieces of research attempting to verify assumptions behind large-scale interventions already made and planned in order to slow the rate of dam siltation to allow water export to South Africa. Here we discuss what our study helps to clarify, and which issues it reveals still need to be clarified.

*Do shrubs and grasses compete in the Lesotho highland rangelands?*

We find no evidence that grasses and shrubs compete in the Lesotho rangelands. Shrubs overshadow grasses most of the time once the shrubs grow over 10 cm, but higher presence of shrubs did not reduce grasses biomass. Shrub seedlings can be easily overwhelmed by surrounding grasses of a similar height. As shrubs age, they also take on a morphology with internal gaps that allows grasses to grow inside the shrub canopy space (Fig. 3). Increasing grazing pressure significantly reduced grasses biomass, as might be expected. Unexpectedly, we found that shrubs may compete with forbs (or some forbs). An increasing shrubs abundance was correlated with a reduction in forb abundance. With increasing time since shrub removal, the richness of the total plant community, dominated by forbs, doubled. This suggests that shrubs may prevent the establishment of a rich forb community.

*Do shrubs and livestock increase or decrease soil moisture *vs* run-off?*

Multiple interviewees including local researchers, conservation workers, herders and farmers claimed that shrubs increase the ability of water to filter into the soil, due to their branching and root structure which could direct water into the soil. Although shrub shoot and root architecture may affect soil water infiltration (Zhang et al., 2020), the infiltration process is likely to also be affected by changes in soil porosity under woody plant canopies and litter layers (Daryanto, Eldridge & Wang, 2013; Liu et al., 2022). The rate of water infiltration is also influenced by rainfall intensity, slope, soil bulk density, porosity, texture, and soil moisture (Harisuseno, Khaeruddin & Haribowo, 2019). Although dry soils are hydrophobic and will initially retard infiltration (Francis et al., 2007), in hydrophilic or wet soils, infiltration rate will decline as the soil becomes saturated during a rain event, and on steeper slopes (Harisuseno, Khaeruddin & Haribowo, 2019). Post-fire soil repellency will also reduce infiltration rates (Noske et al., 2016). The local description of shrub-water interactions might be understood as a way of saying that there is lots of moisture in the soil around or below shrubs, through an assumption about the mechanism resulting in this state which may or may not be correct or fully correct. We found that soil volumetric water content was explained by shrub number and a soil hardness-elevation interaction, which could be consistent with this interpretation of the local claim about the effect of shrubs on water. The local claim could also be taken as a not fully accurate description of the process of soil water infiltration which is often discussed by conservation workers collaborating with Savory method consultants. The large root system of shrubs is also claimed by many of our interviewees to be the reason that shrubs can survive during periods of drought. Thus, we might expect shrubs to both survive droughts due to access to lower soil water profiles, while also increasing surface soil volumetric water content potentially through a combination of canopy shade and a root structure that extracts water *via* evapotranspiration from a lower soil profile.

Conservationists working with Savory method consultants claim that the combination of livestock herding and shrubs reduces water infiltration, causing increased erosion from run-off. Since shrub encroachment alone is generally expected to decrease run-off (*e.g.*, Nie et al., 2012) they are presumably making reference to a complex interaction between livestock, shrubs, and infiltration dynamics (*e.g.*, Soliveres & Eldridge, 2014; Eldridge, Wang & Ruiz-Colmenero, 2015; Cai et al., 2021). Our own results imply that livestock may also have a role in modulating the observed interaction between soil VWC and soil hardness × elevation. This relationship may be driven by livestock pressure, since, averaging across seasonal grazing patterns, livestock density is generally highest at low elevations near towns, and lowest in the highlands. High livestock densities at low elevation may increase soil hardness and may also do so to a lesser extent at higher elevations. In this study, which focuses on ecological outcomes at a point in time, we could not measure dynamic water infiltration processes, but soil moisture is one factor that can affect water infiltration (Harisuseno, Khaeruddin & Haribowo, 2019). Consquently, our results raise the question for future work as to whether observed increased surface soil volumetric water under shrubs can increase infiltration rates.

Livestock trampling typically causes soil compaction and disrupts soil surfaces, which can reduce soil water infiltration (Warren et al., 1986; Dunne, Western & Dietrich, 2011; Daryanto, Eldridge & Wang, 2013). Thus a second question for further research is how the two factors of soil disruption and surface soil moisture interact to create a net soil water infiltration outcome. Ultimately, this is relevant for management if it can be shown to control the rate of erosion.

Finally, it is also possible that shrubs are associated with low soil water infiltration, high run-off and increased erosion because fire, which is set in areas with shrubs, can have these effects (Noske et al., 2016). Specifically, according to our interviewees, herders sometimes set fires to promote grasses regrowth in the short term, which is claimed by the same people to also increase shrub encroachment. Thus, the cause of runoff and erosion—fire, rather than fire-stimulated shrub regrowth—could, we suggest, be observationally confounded. We did not find any effect of recent fire on shrub cover or VWC, but this is undoubtedly because despite our efforts to include sites with a range of management histories, we had only two sites with recent fire in our sample. This may reflect its illegality. However, despite being currently illegal, the use of fire certainly forms part of the set of management tools the effects of which local people have ecological knowledge about. We noticed differences in perspective, however, regarding the dangers and benefits of pasture-burning. Whereas herders generally saw it as a useful tool, conservation workers, some older men, and women saw it as a cause of soil erosion. It is possible that further studies focusing on the role of fire as a management tool might explain why many local researchers, conservationists, and herders associate shrub increase with erosion.

*Does shrub removal decrease erosion?*

Although shrub removal did increase forb abundance and richness, it is not totally clear why shrub removal should decrease erosion. Erosion is expected to increase with shrub presence through the mechanism of reducing grasses cover and forming a bare soil matrix under and between shrubs, which should reduce water infiltration, increase runoff during precipitation, and lead to erosion (*e.g.*, Wainwright, Parsons & Abrahams, 2000; Turnbull et al., 2008; Manjoro, Kakembo & Rowntree, 2012; Brazier et al., 2014). We found no evidence for shrubs-grasses competition, and grasses occurred under and between shrubs in areas with shrubs. The claim that erosion is caused by shrub encroachment depends on a corollary claim that shrubs compete with and reduce grasses cover (*e.g.*, Wainwright, Parsons & Abrahams, 2000; Turnbull et al., 2008; Manjoro, Kakembo & Rowntree, 2012; Brazier et al., 2014). Shrubs cause erosion not by their mere presence, but by reducing grasses cover; grasses cover prevents erosion (*e.g.*, Wainwright, Parsons & Abrahams, 2000; Turnbull et al., 2008; Manjoro, Kakembo & Rowntree, 2012; Brazier et al., 2014). Because we show here that shrubs do not compete with grasses, our data does not support the interpretation that increases in shrubs would have caused increased erosion by reducing grasses cover. We also note that we did not observe sufficient rills or gullies, which would be evidence of erosion by water, at our study sites to systematically record their presence (compare examples of significant presence of rills or gullies in Manjoro, Kakembo & Rowntree (2012), Boardman et al. (2017)). Further studies would also be necessary to determine whether erosion rates have in fact increased in the Lesotho highlands, and how this relates to rainfall events (compare *e.g.*, Turnbull et al., 2008; Brazier et al., 2014).

Our model explaining VWC measures in soil also suggested that one factor affecting increasing soil moisture is an increase in shrub cover, which has also been observed in other shrub systems as a result of stem flow, hydraulic lift, and/or reduction in evaporation rates under the canopy (*e.g.*, Pariente, 2002; Wang et al., 2011; Zou et al., 2005). Shrub-associated patches of soil moisture in semi-arid habitats like this one may increase initial water absorption during precipitation events (Francis et al., 2007). Removing shrubs would reduce this effect and would temporarily create, rather than reduce, the bare soil patches that may cause water runoff and erosion. Further studies of shrub removal explicitly measuring erosion outcomes in a controlled experiment would be useful.

*What is the role of forbs in perceptions of rangeland degradation?*

Shrubs are seen as indicating rangeland degradation, which in addition to describing erosion, describes loss of high-quality livestock forage (Belayneh & Tessema, 2017). However, our data suggest that shrub abundance as a sign of loss of high-quality forage is being misread or unclearly represented. We find that in the Lesotho highlands, high shrub abundance or shrub establishment is not a sign of decrease in grass forage. However, it is a sign of decrease in forbs. We suggest that what rangeland degradation actually consists of, if it consists of something, is a loss of forb abundance and richness.

This study did not set out to prove that forbs have a key role in rangeland degradation. Our finding that forbs, not grasses, are reduced in the presence of shrubs was unexpected. Here we consider how a reduction in forbs but not grasses might be able to explain the perception of rangeland degradation, which should also be considered a subject for further study in the Lesotho highlands context. If livestock show signs of doing poorly and not eating enough in the presence of shrubs, which does not reduce grasses abundance, we suggest that this points to impacts of not eating sufficient forbs, which may help with particular nutrients, improve digestion, and allow self-medication (Provenza et al., 2003; Villalba & Provenza, 2007). Provenza et al. (2003) discuss how it is important both for livestock to be exposed to a variety of plants as they learn to forage, and to have access to different plants across time and space. Decreases in forbs along with increases in shrubs could reduce these opportunities. It may also prevent detoxification of secondary metabolites and increase satiation, thus reducing intake of forage in general (Provenza et al., 2003). A decrease in forbs may be easy to overlook from a herder or land manager perspective, since they are not the main bulk of livestock foraging. However, because it is also the case that forbs are not always included in ecological theory about shrub encroachment especially in rangeland contexts, we did not spend adequate time asking herders and land managers specifically about them. It is thus possible that they have local knowledge relative to forbs that might help to interpret our empirical findings, but which we did not learn about. Potentially, the transformation of the habitat to a more shrub-shaded and slightly more mesic condition might disfavour a number of forb species that may be sun- and arid-adapted, accounting for the observed reduction in forbs in current conditions of shrub encroachment.

*Alternative approaches to management of the Lesotho highland rangelands?*

An alternative approach to shrub removal with a more favorable balance of outcomes might be to seed rangelands, especially in areas where shrubs were recently removed or in areas with elderly shrub populations, with native or endemic nitrogen-fixing forbs. Grasses (and forbs) can allocate more energy to fast growth in resource-rich situations than can woody shrubs (Hunt & Cornelissen, 1997), which can be especially critical during establishment. Grasses have been observed to be favoured over shrubs in high-nutrient soils in heathlands, acacia savannas and other systems (Berendse, Schmitz & de Visser, 1994; Ward, 2005; Suttle, Thomsen & Power, 2007; but see Brown, Scanlan & McIvor, 1998). We thus hypothesize that, through return of N to the soil from native N-fixing forbs, shrub establishment might decrease and grasses and forbs abundances increase. Provenza et al. (2003) also suggest that, depending on the secondary metabolites found in the shrubs, sewing high-protein forbs (such as nitrogen-fixers) and/or high-energy forage within rangelands can increase livestock dietary mixing, favoring the suppression of dominating species (including shrubs) *via* herbivory.

## Conclusions

In sum, although shrub encroachment is considered to be an important problem facing rangeland ecosystems globally, this case study shows that it is still incompletely understood, both in regard to its impacts on diversity, productivity, and soil properties, as well as its causes. The balance of relevant factors affecting shrubs may be different across different shrub species, different ecosystems, or sites with different disturbance or management histories. To better understand how to interpret and manage particular sites, a clearer picture of how each component of shrubs-grasses-forbs systems interacts is warranted. This study has sought to bring some clarity to the discussion by emphasizing the role of forbs in plant competition and livestock production. Equating shrub presence with declines in available forage may be hasty, and greater attention should be paid to the role of forbs in livestock diets and ecological interactions. Shrubs can have positive effects on rangeland productivity, given their role in facilitating precipitation infiltration into the soil, and their removal might have negative consequences that should be better understood.

## Supplemental Information

10.7717/peerj.13597/supp-1 Supplemental Information 1 Field survey data for analysis in R. Data on vegetation, soil, and grazing status are provided for each site that we sampled.

10.7717/peerj.13597/supp-2 Supplemental Information 2 Data on grass-shrub co-presence and plant height. Data are presented on the co-presence of grasses and shrubs in relation to plant height and grazing status.

10.7717/peerj.13597/supp-3 Supplemental Information 3 Data on species richness. Data are provided on species identity and richness for each sampled quadrat.

## Additional Information and Declarations

### Data Availability

The following information was supplied regarding data availability: The raw data is available at OSF: Root-Bernstein, Meredith. 2022. “Does Shrub Encroachment Reduce Foraging Grass Abundance through Plant-Plant Competition in Lesotho Mountain Rangelands?” OSF. July 1. osf.io/6tpwb.
